# Enhancement of wildlife disease surveillance using multiplex quantitative PCR: development of qPCR assays for major pathogens in UK squirrel populations

**DOI:** 10.1007/s10344-016-1031-z

**Published:** 2016-07-28

**Authors:** Timothy D. Dale, Phillip C. Watts, David Jones, Kieran Pounder, David J. Everest, Michael E. Begon, Julian Chantrey

**Affiliations:** 1 Institute of Integrative Biology, University of Liverpool, Biosciences Building, Crown Street, Liverpool, L69 7ZB UK; 2 Department of Ecology, University of Oulu, 90014 Oulu, Finland; 3 Institute of Infection and Global Health, University of Liverpool, Liverpool, UK; 4 Animal and Plant Health Agency, Weybridge, New Haw, Addlestone, Surrey, KT15 3NB UK

**Keywords:** Adenovirus, Endogenous, Internal, Control, Squirrelpox, Virus

## Abstract

Rapid development in polymerase chain reaction (PCR) technology has revolutionised the speed and accuracy of many diagnostic assays. However, comparatively few wildlife epidemiological studies use quantitative PCR (qPCR) for pathogen detection, even fewer employ an internal control, to ensure confidence in negative results, and PCR’s ability to multiplex and therefore detect several targets in a single reaction is underutilised. Here, we describe the development of two multiplex qPCR assays for the red and grey squirrel that detect the pathogens squirrelpox virus (SQPV) and adenovirus in squirrels (SADV), both of which cause mortality in the red squirrel. Both assays use a section of the squirrel phosphoglycerate kinase gene as an endogenous internal control that identifies and compensates for both, inadequate sampling or PCR inhibition. Tests on infected squirrel tissue demonstrate that simple swab samples (particularly from distal antebrachial skin) are sufficient to detect and identify the relative quantity of SQPV DNA in both squirrel species, while rectal swabs and blood cell pellets can be used to reliably indicate SADV infection. These assays are sensitive and specific with an endogenous internal control providing confidence in negative results and allowing comparison across laboratories. Using such assays should prove advantageous in wildlife studies with limited resources while allowing the maximum data yield.

**Electronic supplementary material:**

The online version of this article (doi:10.1007/s10344-016-1031-z) contains supplementary material, which is available to authorized users.

## Introduction

Effective wildlife disease surveillance is important, with links between wildlife and pathogens relevant to human and domestic animal health being commonly discovered, e.g. *Echinococcus multilocularis* (Hegglin et al. 2015), SARS coronavirus (Bell and Roberton 2004), avian influenza (Fouchier et al. 2004), hantavirus (Watson et al. 2014) and Ebola (Leroy et al. 2005). Wildlife disease surveillance thus forms an essential part of the ‘One Health’ theme (Thompson 2013), with studies providing an opportunity to both benefit human health and the wildlife species in question (Jenkins et al. 2015). To minimise transmission of pathogens between wildlife and livestock or humans, it is necessary to understand the species’ ecology and pathogen transmission pathways (Boadella et al. 2011; Delahay et al. 2009). Any investigation of host-pathogen dynamics relies on a sensitive and specific detection assay (Boadella et al. 2011), and yet application of current molecular techniques to wildlife disease epidemiology has been slow in comparison with studies of humans and livestock (Benton et al. 2015).

Contemporary diagnostic tests for many pathogens rely on polymerase chain reaction (PCR) as assays often yield excellent sensitivity and specificity (for both fresh and archived samples) and in less time than many traditional assays (Cai et al. 2014). In particular, quantitative PCR (qPCR) combines high-throughput sample processing with increased sensitivity and reduced potential for contamination (Klein 2002). However, PCR can be prone to inhibition in all formats (Brankatschk et al. 2012; Schrader et al. 2012) and quality control is therefore vital during assay development and processing (Borst et al. 2004; Burkardt 2000; Lo and Chan 2006). One key quality control measure is to include an internal control (IC) to minimise occurrence of false-negative results. ICs that consist of a known DNA sequence, added to the sample prior to or post-DNA purification (exogenous IC), are often convenient (e.g. in commercial kits) but may impair assay sensitivity (Cai et al. 2014). Alternatively, a region of the host’s genomic DNA (reference gene) can be used as an IC (endogenous IC) to verify the absence of PCR inhibitors during sample processing; using an endogenous control prevents the need to add foreign DNA to the qPCR and allows calculation of relative quantity of target DNA. Internal endogenous controls are frequently used in gene expression studies but rarely used in pathogen detection assays. Multiplex PCRs can be used to detect simultaneously the target and endogenous IC sequences and thus save time and costs (Cai et al. 2014; Elnifro et al. 2000).

Despite the putative advantages of qPCR assays, only 35 % (48/136) of reviewed studies of wildlife disease surveillance published since 2011 have used qPCR; furthermore, only 8 % (7/88) and 11 % (5/45) of conventional PCR and qPCR studies, respectively, used an IC. Use of a multiplex reaction was similarly limited, accounting for only 14 % (12/88) and 10 % (4/39), respectively, of wildlife disease surveillance studies. The implication is that contemporary techniques in molecular epidemiology are underutilised in wildlife disease surveillance (see also Benton et al. 2015).

The Eurasian red squirrel (*Sciurus vulgaris*) in the UK, has suffered a severe reduction in its abundance and distribution (Bosch and Lurz 2012), coinciding with the introduction of the non-native Eastern grey squirrel (*Sciurus carolinensis*) in the 1900s (Gurnell and Pepper 1993; Usher et al. 1992). A combination of modelling (Rushton et al. 2006; Tompkins et al. 2003), experimental infection studies (Tompkins et al. 2002) and field data (Chantrey et al. 2014) indicates that a major part of the decline of UK red squirrel populations is due to infection by squirrelpox virus (SQPV). This pathogen is thought to have been introduced with the grey squirrel, a theory supported by a high prevalence of exposure despite an absence of clinical disease (Bruemmer et al. 2010; Sainsbury et al. 2000), an association between seropositive grey squirrels and the appearance of clinical cases of squirrelpox in red squirrels (McInnes et al. 2009, 2013; Sainsbury et al. 2008) and an association of the pathogen in both squirrel species during an outbreak of disease in red squirrels (Chantrey et al. 2014). Programmes to reintroduce red squirrels in the UK have been hampered by disease: by SQPV in Norfolk (Carroll et al. 2009) and adenovirus in squirrels (SADV) in other areas of the UK (Everest et al. 2008, 2012b; Martinez-Jimenez et al. 2011). SADV appears to cause mortality in wild red squirrels (Duff et al. 2007; Everest et al. 2012a), although the impact on a population scale is largely unknown with subclinical infections also reported (Everest et al. 2014).

It is important that studies of this system are based on reliable and accurate diagnostics. SQPV infection status was initially determined using transmission electron microscopy (TEM) (Scott and Keymer 1981), but more recently conventional PCR (McInnes et al. 2009) and qPCR (Atkin et al. 2010; Fiegna et al. 2016) assays have been developed. As grey squirrels appear asymptomatic, lower infection loads are recorded, and thus it is essential to use the more sensitive qPCR assays when screening for SQPV presence as part of a wildlife disease management programme for red squirrels. For SADV infection, confirmation has relied on either TEM (Everest et al. 2008) or conventional qualitative PCR assays (Everest et al. 2009b). No assay for either pathogen has incorporated an IC (either exogenous or endogenous). Additionally, the use of ICs to monitor squirrel pathogens is essential because staphylococcal exudative dermatitis (Simpson et al. 2010, 2013a) and rotaviral enteritis (Everest et al. 2009a) show similar lesions to SQPV and SADV, respectively. This presents a clear opportunity for misdiagnosis (in the absence of other diagnostic tests) if the pathogen assay reports a false negative, for example due to unrecognised presence of PCR inhibitors.

To encourage other wildlife disease surveillance studies to develop qPCR assays with the potential to detect multiple pathogens along with an IC to assess qPCR quality, we describe the development of two qPCR assays that are capable of detecting both SQPV and SADV in a sample from either red or grey squirrels. These assays thus present substantial improvements on current methods to detect these agents and will facilitate our understanding of the epidemiology of squirrel pathogens.

## Methods

All qPCR reactions were carried out using white 96-well plates sealed with optical clear caps (Primer Design, Southampton, UK) and were run in a LightCycler® 480 II real-time cycler (Roche, Welwyn Garden City, UK), unless otherwise stated.

### Endogenous control gene selection

Potential gene targets for the endogenous control were identified from studies on other mammals (de Jonge et al. 2007). BLASTn (Altschul et al. 1990) was used to align gene sequences for the endogenous control candidates, using sequence data from the mouse (*Mus musculus*) and the 13-lined ground squirrel (*Ictidomys tridecemlineatus*), the only available genome from the *Sciuridae* family (GenBank Accession number AGTP01000001-01024510) (Benson et al. 2010). Conserved regions between the mouse and 13-lined ground squirrel were assumed to be good candidates for sequence conservation between red and grey squirrels. Those conserved regions were then sequenced to investigate their suitability to amplify host DNA in a qPCR (as described in Online Resource S1).

### Multiplex PCR design

AlleleID® v.7 (Premier Biosoft, CA, USA) was used to design primers and probes for qPCR against the (1) SQPV G8R gene (GenBank NC022563), (2) SADV DNA polymerase gene (GenBank JN205244), the targets for published assays (Atkin et al. 2010; Everest et al. 2009b), and (3) the two endogenous IC sequences. A BLASTn search (Altschul et al. 1990) was used to confirm that primers and probes would not bind to non-target regions. Primers were tested with DNA from squirrels and with known positive and negative DNA templates. For SQPV, positive controls were those previously tested positive using the assay developed by Atkin et al. (2010); for SADV, positive controls were material that had shown a high SADV load by TEM. Reactions were initially run and optimised as uniplex reactions using Precision™ SYBR® Green Master Mix (Primer Design, Southampton, UK) using the reaction constituents and cycle parameters described in Online Resource S1. The optimum qPCR annealing temperature (*T*_a_) was determined first using a 55–65 °C temperature gradient, and amplicons were assessed also for specificity using a melt curve analysis (a single melt point was shown within the temperature predicted by the PCR design software). Primer concentrations were varied between 50 and 500 nM, with the primer concentration that gave the lowest crossing point (*C*_p_) without the appearance of additional peaks in the no-template control (NTC) on the melt curve considered optimum.

The fluorescent reporter dyes were FAM (endogenous control), Texas Red (SQPV) and Cy5 (SADV), with quenchers BHQ-1, BHQ-2 and BHQ-3, respectively, chosen to ensure minimal spectral overlap (Marras 2006). Hydrolysis probe sequences were designed concurrently with the primers using AlleleID® v.7 (Premier Biosoft, CA, USA) and were trialled first in uniplex using Precision™ Master Mix without SYBR® Green (Primer Design, Southampton, UK). Optimal probe concentrations were determined as for primer concentration optimisation. The optimal amount of sample (unknown quantity of target) genomic DNA per reaction was defined as the largest amount of total DNA that showed a low *C*_p_ across all assays but which avoided excessive dilution of samples (thereby not affecting the analytical sensitivity of the assay).

An initial assessment of the ability of the assays to be multiplexed was carried out by comparing *C*_p_ values obtained with the same DNA templates but with primers used in uniplex, duplex and triplex reactions. A mixture of previously identified positive samples was used to create a DNA template that was positive for the IC and both of the target genes. Multiplex qPCRs were run as 20 μl final reaction volumes that contained 4 μl 5 × QuantiTect® virus Master Mix (Qiagen, Manchester, UK), appropriate amounts of each primer and probe (Table 1) and ca. 50 ng template DNA. Thermal cycling conditions were 5 min at 95 °C, followed by 50 cycles of 15 s at 95 °C and 75 s at 60 C. PCR efficiencies of reliable multiplex qPCRs were assessed using serial dilution of standards (10^7^–10^2^ copies and 5^7^–5^2^ copies) with five reactions per dilution. As neither virus is readily isolated in cell culture, we modified the qPCR protocol of O’Callaghan and Fenech (2011) to quantify the amount of target DNA. Briefly, known quantities of synthetic oligonucleotides (Eurofins MWG Operon, Edersberg, Germany, or Integrated DNA Technologies, Leuven, Belgium) (see Online Resource S2) for calculations to determine number of copies based on molecular weight of the oligonucleotides), whose single-stranded DNA sequences are those of the control and target genes that binds the probe, are used to create a standard curve against which the amount of target DNA in the unknown samples can be calculated (O’Callaghan and Fenech 2011). With no gold standard assay for either target and therefore no confidence about a ‘truly negative’ sample, Lambda (*λ*) DNA (Fisher Scientific, Loughborough, UK) was added to the serial dilutions to ensure the same quantity of DNA overall in each qPCR (Online Resource S3). Only multiplex qPCRs with efficiencies between 90 and 110 % for all assays were retained.

**Table 1:** Primer and probe sequences for the grey and red squirrel pathogen assays with optimised primer and probe concentrations and annealing temperature (*T*_a_) range showing maximum band intensity, with selected final *T*_a_ (60 °C)

Multiplex set	Target	Primer (L/R)/probe (P)	Sequence 5′–>3′	Concentration (nM)		*T*_a_ range (°C) [selected optimal]
				Uniplex/duplex	Triplex	
Grey	SQPV	L R P	CATCGACCAGAAGAAGTC GCTGATGCACTTGATGAA TexR-CGTGTTCAACTTCCACCTCTACG-BHQ2	200 300 300	200 200 200	55–65 [60]
	ADV	L R P	CGGGAATCTTTACAATCG TGTCCATGTTAGTCTTCC Cy5-AAGAATGGACCGACACATTGCC-BHQ3	300 300 300	200 200 200	57–63 [60]
	PGK	L R P	GGTCTATTATCCTGTTGGA CTGGTTTGGAAAGTGAAG FAM-TACTTCGGCTGACTCGGCTT-BHQ1	300 200 400	200 200 200	58–62 [60]
Red	SQPV	L R P	TGGGTCTTCGCATAAAAC GACCTCTTCCGAGAACTC TexR-CGTCACTATCTGCCTCAACCG-BHQ2	300 200 200	200 200 200	55–62 [60]
	ADV	L R P	TCCGGGAATCTTTACAATC CAGAGATTCATTTGTCCATG Cy5-AGAATGGACCGACACATTGCC-BHQ3	300 300 300	200 200 200	55–65 [60]
	PGK	L R P	CTGGGAACAAGGTGAAAG TCATCAYTCACATAGACATCC FAM-CGAGCCAGCCAAAGTAGAAGC-BHQ1	300 200 300	200 200 200	57–62 [60]

The linear ranges of detection for the assays were determined using serial dilutions of the standards from 10^13^ to 10 copies per reaction, and the limit of detection was established with twofold dilutions of standards from 100 to 1.0625 copies per reaction. Repeatability of the limit of detection was confirmed by making a further six triplicate reactions and further testing with the determined limit of detection with high levels (1.0 × 10^7^ copies per reaction) of the ‘other’ two assayed genes.

Optimised assays were tested for pathogen specificity by seeding six qPCR reactions with DNA extracted from tissue infected with monkeypox virus (two tissue samples) and cowpox virus (two tissue samples) and individual cell culture isolates that contained canine adenovirus 1 and 2. The results of the new assays were compared with the SQPV PCR assay (Atkin et al. 2010) and TEM methods used to identify SADV (Everest et al. 2008). In the absence of a gold standard for both target pathogens, a test of agreement (Pfeiffer 2010) was performed to compare whether the assays similarly reported presence or absence of virus. When comparing qPCR assays, the quantitative values were investigated for correlation using a Pearson’s correlation. All statistical analyses (including generating figures) were performed using R v.2.15.1 (R Core Team 2012). Where quoted, all confidence intervals (CI) are at the 95 % level.

### Reaction plate setup

All unknown sample extracts were run in triplicate with one NTC for each sample using the optimised qPCR protocol. Each reaction plate contained (1) a positive control for the target genes, (2) a standardised calibrator sample (1 × 10^7^ copies of reference and target genes made up to 200 ng total DNA using *λ* DNA) and (3) a triplicate of a standard of known quantities of all assay genes. See Online Resource S4 for the layout of a typical sample plate. One standard curve (imported during software analysis) was used for analysis of several qPCR plates, rather than making a standard curve for each plate (allowing more samples per plate); this allowed the standard within the plate to act as a reference on the standard curve while the calibrator acted as a reference for inter-plate variation (see normalisation calculation below). The mean *C*_p_ value was converted to a copy number and a normalised relative quantitative ratio (RQ) of the target gene to reference gene was then calculated using the following equation:

$$ \mathrm{R}\mathrm{Q}={\left(\frac{T}{R}\right)}_{\mathrm{sample}}\ /{\left(\frac{T}{R}\right)}_{\mathrm{calibrator}} $$

Where,

RQ: Relative quantification ratio
*T*: Target copy number and
*R*: Reference gene copy number.

A sample was considered positive if two or three (of three) replicates amplified the target and there was no amplification in either of the NTC or the no-template batch extract. If only one reaction showed a positive amplification the sample was re-run and if the result was repeated the sample was classed as inconclusive.

### Red and grey squirrel sample trial

Multiplex assay performance was further quantified using necropsy tissue samples with the aim of quantifying the infection load of tissues from red squirrel carcasses found during autumn 2011. Some squirrels were suspected to have SQPV or SADV based on gross lesions (skin ulcers or enteritis, respectively), while three red squirrels had no sign of disease (i.e. road casualties) and three red squirrels were selected on the basis that they had gross lesions not traditionally associated with either SQPV or SADV (skin or gastrointestinal inflammation, respectively). As grey squirrels rarely show clinical signs for either infection, 16 carcases were selected at random from samples submitted as part of a grey squirrel control programme (during autumn 2011). All squirrels had a systematic necropsy examination. Swabs (Dacron tip, Fisher Scientific UK Ltd., Loughborough, UK) were taken from sites believed to have a predilection for SQPV infection (i.e. oral/lip, eyelid and/or arm vibrissae) (Atkin et al. 2010) or SADV enteric infection (rectum). Corresponding skin samples were taken, along with flank and chest (vibrissae) skin samples for grey squirrels and nose, ear, digit, inguinal and genital skin samples for red squirrels. Submandibular lymph node, lung, heart, liver, kidney, spleen, stomach, small intestine (duodenum and jejunum), large intestine (caecum and colon), faeces, skeletal muscle and brain were all sampled. Blood was collected post-mortem and serum was separated from the cell pellet by centrifugation (13,000 rpm for 10 min). All samples were stored at −20 °C until DNA purification using a DNeasy® blood and tissue kit (Qiagen, Manchester, UK) and the manufacturer’s protocol. DNA was extracted from swabs using protocol (a) in the Online Resource S5. DNA was isolated from the blood cell pellet using a FlexiGene DNA kit (Qiagen, Manchester, UK) following the protocol (b) in the Online Resource S5. A ‘no sample’ DNA extraction was completed in tandem with every batch of extractions to monitor potential contamination during DNA extractions.

## Results

### Endogenous control gene selection

The results of the trials to identify the best endogenous ICs are provided in the Online Resource S6 (Tables S6.1 and S6.2): a section of the squirrel phosphoglycerate kinase (PGK) gene was identified as an appropriate endogenous IC for both squirrel species.

### Assay optimisation

**Fig. 1:**
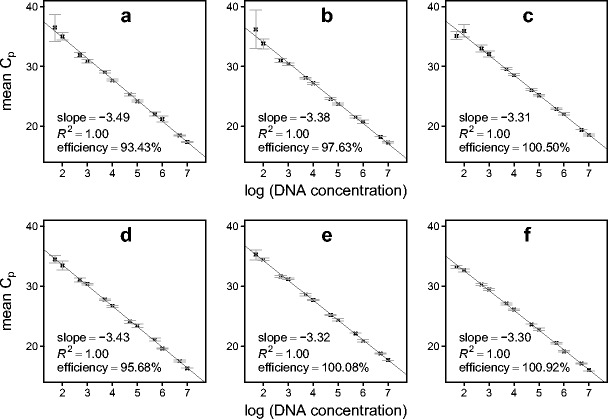
Standard curves produced during triplex qPCR of serially diluted standards for the grey (**a**–**c**) and red (**d**–**f**) squirrel multiplex virus panel (200 nM concentrations of primers and probe). **a**, **d** PGK (control). **b**, **e** SQPV. **c**, **f** SADV

Best multiplex primer and probe panels were derived from AlleleID® (Table 1; see Online Resource S7, Fig. S7.1 for positions on control/target genes); secondary structures (hairpins, homo- and hetero-dimerization) of primers, probes and templates were assessed within the software used. All undesirable secondary structures had a delta G (Δ*G*) >−2 and melting temperature (*T*_m_) at least 30 °C less than the chosen *T*_a_. These suggested inhibitory secondary structures at the reaction temperature were highly unlikely to form and thus have little detrimental effect on the desired PCR reaction. PCR product sequences were confirmed to be the same as predicted target sequences (Online Resource S7, Fig. S7.2–7.7). Brief comparisons between SYBR® green and hydrolysis probe methods demonstrated that both chemistries yielded comparable values of *C*_p_. Moreover, completing the assays as either duplexes or triplexes had little impact on *C*_p_ values. (A summary of these results is provided in the Online Resource S8, Fig. S8.1). Optimal primer/probe concentrations varied between 200 and 400 nm for uniplex reactions (Table 1). While satisfactory efficiencies (i.e. *E* >90 % but <110 %) with duplex reactions were achieved using the same primer/probe concentrations as for uniplex reactions (see Online Resource S8, Fig. S8.2–3 for standard curves), triplex reactions required primers and probes to be of equal concentration (200 nM; as per the manufacturer’s recommendation) to achieve reaction efficiencies comparable to those recorded in duplex reactions (Fig. 1). All assays had maximised amplification over a 5–10 °C range in *T*_a_, with a *T*_a_ of 60 °C acceptable for all assays (Table 1). A total of 200 ng of total DNA per reaction provided the best compromise between obtaining a low *C*_p_ value and maintaining assay sensitivity for both multiplexes.

### Assay validation

#### Molecular specificity

Non-specific binding was not evident (using agarose gel electrophoresis of PCR products) in samples assayed in uniplex, duplex or triplex analyses. That all assays amplified the correct target region was confirmed by Sanger sequencing (Online Resource S7, Fig. S7.2–S7.7). The assays appear specific to the intended squirrel pathogens, as neither SQPV nor SADV qPCR assays amplified (0/6 reactions) a product in qPCRs seeded with either cowpox, monkeypox or canine adenovirus 1 or 2 DNA.

#### Molecular sensitivity

The linear range of the assays between the *C*_p_ value and the log of the target DNA for qPCRs was similar across all assays (Online Resource S9, Fig. S9.1), containing between 10^2^ and 10^10^ copies of the target gene. The consistent, reliable limits of detection for the qPCR assays in both red and grey squirrels were estimated to be about 12 (PGK) or 6 (SQPV and SADV) copies per reaction.

#### Comparison to existing assays

Our assay detected SQPV with comparable results to the Atkin et al. (2010) SYBR® green assay, showing good qualitative agreement; *k* value (agreement) of 0.75 (*n* = 7) and 1.00 (*n* = 12) for grey and red squirrel assays, respectively. In comparing the quantitative values (i.e. *C*_p_ values) of the developed SQPV assays and the Atkin et al. (2010) assay, strong correlation was witnessed (*r* = 1.00 and 0.99 for grey and red squirrel assays, respectively). By contrast, we found only moderate agreement between qualitative results of SADV detected in tissues by TEM and our assay results (*k* value = 0.39, *n* = 15; red squirrel SADV assay). In fact, none of the grey squirrel tissues tested (*n* = 5) showed evidence of SADV viral particles by TEM, which was not surprising due to the asymptomatic nature of the infection in the grey squirrel. By contrast, the *k* value between the developed red and grey squirrel SADV assays was 1 and it showed a strong correlation between quantitative results (*r* = 1.00, *n* = 15).

Our uniplex and multiplex assays gave the same information about whether samples were positive or negative (*k* value = 1.00 (*n* = 8 per assay)) and displayed high correlations in the absolute quantification (i.e. *C*_p_ values) of viral load (*r* = 0.82, 0.98 and 0.85 for grey squirrel assays and *r* = 0.98, 0.98 and 1.00 for red squirrel assays, with correlations displayed for tests of PGK, SQPV and ADV, respectively). When viral loads were calculated as RQ values, the correlation coefficients for comparison of uniplex and multiplex assays were *r* = 1.00 and 1.00 (*n* = 8) for grey squirrel SQPV and SADV assays and *r* = 0.85 and 1.00 (*n* = 8) for red squirrel SQPV and SADV assays, respectively.

#### Grey squirrel sample trial

All sample types investigated contained adequate amounts of squirrel DNA for amplification of the IC, with even those samples with expected low yields of squirrel DNA (cutaneous swabs, especially those from the arm area) amplifying the IC (see absolute quantification results, Online Resource S10, Fig. S10.1).

**Fig. 2:**
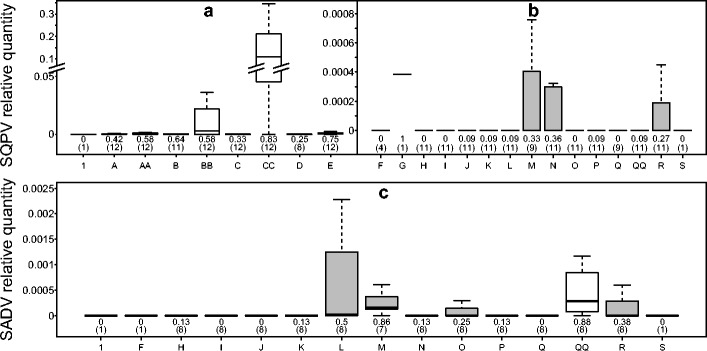
Relative quantification (RQ) values obtained as a function of sample type for SQPV skin samples (**a**), tissue samples (**b**) and SADV (**c**) for positive grey squirrels. Sample types along the *x* axis are defined as follows: *1* lice, *A* lip skin, *AA* oral swab, *B* eyelid skin, *BB* eye swab, *C* arm skin, *CC* arm swab, *D* chest skin, *E* flank skin, *F* submandibular lymph node, *G* thymus, *H* lung, *I* heart, *J* liver, *K* kidney, *L* spleen, *M* blood, *N* stomach, *O* small intestine, *P* large intestine, *Q* faeces, *QQ* rectal swab, *R* skeletal muscle, *S* brain. Tissue samples are shaded in *grey*. Note axis break in *y* axis of (**a**) where axis ticks represent the same interval. Displayed below each boxplot are the number of animals examined (*n*) in parentheses and the proportion of infected individuals that showed a positive result in the defined sample type (*upper number*)

SQPV infections were generally localised to cutaneous areas in grey squirrels (Fig. 2a, b). Arm swabs gave the highest RQ (ratio of target (SQPV) to control gene (PGK)) (mean RQ = 0.18 ± 0.14 CI, *n* = 12) and the greatest proportion of positive results in infected grey squirrels (0.83 (10/12), 0.51–0.97 CI). Eyelid swabs also showed a relatively high RQ of SQPV DNA (mean RQ = 0.029 ± 0.027 CI, *n* = 12), although it appears the area is less frequently infected, with the proportion of positive results from eyelid swabs being lower (0.58 (7/12), 0.29–0.84 CI) than on the arm. Flank skin samples appeared to be commonly infected with the virus in infected individuals (0.75 (9/12), 0.43–0.93 CI) but with a low intensity of infection (RQ mean = 0.004 ± 0.007 CI, *n* = 12). Non-cutaneous samples showed low incidence and intensity of SQPV infection, with blood, stomach and skeletal muscle showing the highest prevalence of the virus in positive animals all ca. 0.3 (Fig. 2b).

SADV infection also appears to be localised in grey squirrels, with the highest level of infection detected in rectal swab samples (RQ mean = 0.0009 ± 0.001 CI, *n* = 8), with 0.88 (7/8, 0.47–0.99 CI) of infected individuals showing presence of the virus on such swabs (Fig. 2c). However, tissue samples from the gastrointestinal tract (i.e. stomach, large intestine and faeces) yielded low numbers of the virus (e.g. small intestine RQ mean = 0.00009 ± 0.0001 CI, *n* = 8) and with only a quarter (0.25 (2/8), 0.05–0.64 CI) of the infected grey squirrels showing a positive result in the small intestinal tissue. Interestingly, values of RQ in the spleen were somewhat higher (RQ mean = 0.0007 ± 0.0009 CI, *n* = 8), with viral presence in half (0.5 (4/8), 0.18–0.82 CI) of the SADV-positive animals. The findings in the spleen are also complemented with a high proportion of viral presence in the blood (0.86 (6/7), 0.42–0.99 CI, *n* = 7) all-be-it with relatively low quantity of virus (RQ mean = 0.0003 ± 0.0002 CI, *n* = 7). All other samples/tissues showed a low prevalence of infection (≤0.13 (1/8), 0.01–0.53 CI) (Fig. 2c).

#### Red squirrel sample trial

Six red squirrels were submitted for post-mortem examination with gross skin lesions consistent with SQPV infection, and seven red squirrels were acquired with abnormalities in the gastrointestinal tract that were compatible with a SADV infection. Of the three squirrels whose mortality was not thought related to SQPV or SADV infection, two had severe pediculosis and the other a severe pneumonia.

As with the grey squirrels, swab samples effectively recovered both target and reference DNA in red squirrels, with only one arm swab failing to isolate squirrel DNA (*n* = 6 for each cutaneous swab site and *n* = 7 for rectal swabs) (see absolute quantification results in the Online Resource S10, Fig. S10.2).

**Fig. 3:**
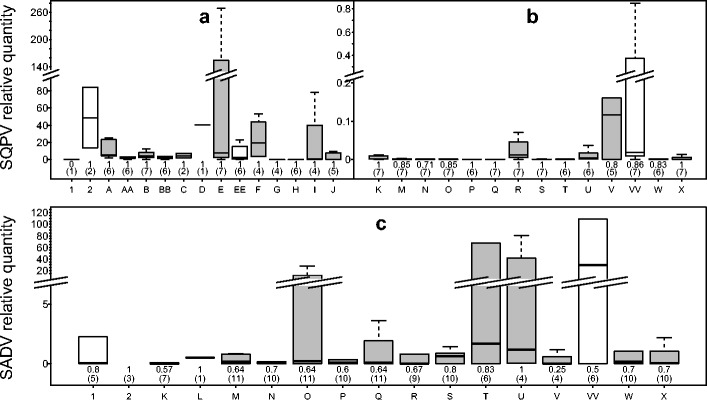
Relative quantification (RQ) values obtained as a function of sample type for SQPV skin samples (**a**), tissue samples (**b**) and SADV (**c**) for positive red squirrels. Sample types along the *x* axis are defined as follows: *1* lice, *2* fleas, *A* lip skin, *AA* oral swab, *B* eyelid skin, *BB* eye swab, *C* ear skin, *D* nose skin, *E* arm skin, *EE* arm swab, *F* digit skin, *G* chest skin, *H* flank skin, *I* inguinal skin, *J* genital skin, *K* submandibular lymph node, *L* thymus, *M* lung, *N* heart, *O* liver, *P* kidney, *Q* spleen, *R* blood, *S* stomach, *T* small intestine, *U* large intestine, *V* faeces, *VV* rectal swab, *W* skeletal muscle, *X* brain. Swab samples are shaded in *grey*. Note axis breaks in *y* axis of all three charts, axis ticks represent the same interval. Displayed below each boxplot are the number of animals examined (*n*) in parentheses and the proportion of infected individuals that showed a positive result in the defined sample type (*upper number*)

Cutaneous samples showed a greater RQ of SQPV DNA than other tissues. Arm skin had the highest RQ of SQPV DNA (RQ mean = 1937 ± 3692 CI, *n* = 7), and digit skin was the second most intensely infected area (RQ mean = 24 ± 24 CI, *n* = 4) (Fig. 3a). Of all cutaneous swabs taken, arm swabs yielded the most SQPV DNA (RQ mean = 29 ± 47 CI, *n* = 6). It appears virus is present over much of the body with all cutaneous swabs and tissue showing 100 % SQPV presence in infected individuals. In contrast to grey squirrels, non-cutaneous samples also showed high infection rates in red squirrels but with somewhat lower infection intensities when compared to cutaneous samples (Fig. 3b). For example, blood showed the highest RQ of the non-cutaneous tissues tested (RQ mean = 0.097 ± 0.16 CI, *n* = 7), while rectal swabs provided the highest recovery of SQPV DNA (RQ mean = 0.23 ± 0.33 CI, *n* = 7); indeed, the recovery of SQPV from rectum was comparable to the relative quantity of SQPV DNA found in faeces (RQ mean = 0.18 ± 0.22 CI, *n* = 5).

Samples from the gastrointestinal tract showed the greatest RQ and the highest proportion of viral presence in infected individuals with rectal swabs showing the highest recovery of SADV DNA (RQ mean = 81.09 ± 99.81 CI, *n* = 6) and small intestine being the tissue with the greatest intensity of infection (RQ mean = 46.91 ± 67.69 CI, *n* = 6) and the highest proportion of infection (0.83 (5/6), 0.37–0.99 CI). Out of all other tissues, the liver showed a proportionately high intensity of infection (RQ mean = 7.04 ± 5.97 CI, *n* = 11). Interestingly, lice showed both a high ratio of SADV to squirrel DNA (RQ mean = 78.24 ± 152.23 CI, *n* = 5) and where present on an infected squirrel the prevalence of SADV infection in lice was high (0.8 (4/5), 0.30–0.99 CI).

## Discussion

Despite the obvious advantages of multiplex PCR assays, they are still relatively underused compared with uniplex PCRs, possibly because of a persistent belief that multiplex assays require heavy investment into their setup and optimisation (Elnifro et al. 2000). The methods used here, however, allowed development of assays that are reliable, analytically sensitive and specific and thus fulfil the required criteria for a diagnostic qPCR assay (Bustin et al. 2009). That we developed multiplex qPCR assays for two squirrel species indicates that developing multiplex qPCR assays for multiple pathogens in other wildlife species should be relatively straightforward. A clear benefit is that, since much of the optimisation and validation is carried out in multiplex, there is relatively little additional work required to develop assays that screen for more than one pathogen; moreover, multiplex reactions increase the amount of data gained from a single sample, and incorporating an endogenous IC into the design provides confidence in the reliability of the (negative) results and allows comparison of results across laboratories. Therefore, any disadvantage of additional initial investment into assay development is quickly outweighed by the greater quantity and quality of data produced.

Overall, the new assays identified similar findings to the previously published methods. Our SQPV assays and the Atkin et al. (2010) SYBR® green assay both identified SQPV as a localised infection in grey squirrels, compared with the generalised, systemic infection in red squirrels. The agreement between available TEM data and our qPCR assays for SADV was moderate (*k* value = 0.39), but this level of agreement is nonetheless superior to that reported in studies using conventional PCR. Grey squirrel SADV results are reasonably comparable to previous studies reporting infection prevalence of spleen and blood samples as 54–57 and 0–7 % (Everest et al. 2014) in comparison to 33 and 50 % reported here. The difference in prevalence of SADV in blood samples may reflect the increased sensitivity of the qPCR assay developed, with the level of SADV in blood generally low, and thus potentially overlooked when screening with conventional PCR. However, differences in selection, geography and time of year may also have been responsible for or contributed to the data obtained. In red squirrels, SADV is believed to be an enteric infection although systemic effects are witnessed, with splenitis commonly reported (Martinez-Jimenez et al. 2011; Sainsbury et al. 2001). This is supported by our data, with the gastrointestinal tract, liver, blood and spleen showing the highest intensities and prevalence of SADV infection.

Our assays offer several advantages over assays for SQPV (Atkin et al. 2010; Fiegna et al. 2016; McInnes et al. 2009) and SADV (Everest et al. 2009b), namely increased specificity (using a hydrolysis probe), sensitivity (using qPCR) and a reduction in the occurrence of false negatives (e.g. insufficient DNA purification and PCR inhibition) via the use of a specific squirrel endogenous IC. In fact, the latter factor proved important in 7 out of 24 faecal samples analysed when screening samples, when, despite apparently adequate amounts of DNA, no amplification for the PGK assay (or either pathogen assay) was recorded. In the absence of an IC, these samples could have been classed as negative and highlight that a differing extraction process is required for faecal samples.

Our qPCR assays provide a new, lower-resource and potentially higher-throughput sampling method. The use of an endogenous IC has allowed the validation of the use of swabs to detect the target pathogens. The level of a pathogen on a swab not only depends on the pathogen abundance at the site sampled but also on the amount of sample effort. However, it is reasonable to assume that this latter factor will also dictate the amount of reference DNA harvested. Hence, the use of an endogenous IC compensates for any variability in sample effort—a potential confounding factor when using swabs to collect samples. However, caution is required when comparing swabbed material with tissue samples. Relative quantification is the ratio of the target viral DNA (SQPV/SADV) to the reference squirrel DNA (PGK). Thus, if the sampling method harvests host cells poorly in comparison to viral particles, then an artificially high viral load may be indicated. This may, in part, explain why arm swabs yielded by far the highest RQ of SQPV in grey squirrels (Fig. 2). Unsurprisingly, arm swabs show a poorer harvest of squirrel DNA in comparison to other sample types (Online Resource S10, Fig. S10.1). Yet, in terms of absolute quantities, arm swabs show a proportionally higher number of DNA copies of the SQPV target gene (Online Resource S10, Fig. S10.1). Therefore, the high levels of SQPV in arm swabs appear genuine, a conclusion supported by the infection levels in skin sampled from the same site in red squirrels (see Figs. 2 and 3).

Due to the apparent localised SQPV infection that grey squirrels displayed (Fig. 2, see also (Atkin et al. 2010), diagnosis in this species can prove problematic particularly as no one single tissue can reliably be used to indicate grey squirrel SQPV infection status. While previous attempts to use swabs to diagnose a SQPV infection in grey squirrels failed to identify infected individuals (Collins et al. 2014), swab samples provided several benefits in this study. Swabs allow multiple sites and a greater sampling area to be surveyed without concern for overloading the sample with host DNA. Thus, a single swab sampling the oral, eye and distal arm areas investigated would identify all infected individuals in this study and is recommended as the sampling method of choice in both squirrel species for SQPV detection alone (Table 2). Adding a rectal swab (potentially combined with the cutaneous swab) and blood would make one confident in detecting the majority of SADV cases while also performing analysis for SQPV. It should be noted, however, that reliability of using swabs depends upon the use of the endogenous IC that indicates adequate harvesting of cells (via amplification of squirrel DNA).

**Table 2:** Recommendations for non-invasive (swab or blood sample) or post-mortem (tissue) samples to detect SQPV and SADV in grey and red squirrels

Squirrel species	Sample method	Pathogen					
		SQPV			SADV		
		Individual sample		Combined samples	Individual sample		Combined samples
Grey	Swab or blood	Arm swab	0.83	1.0 (12/12)	Rectal swab	0.88	1.0 (8/8)
		Oral swab	0.58		Blood	0.86	
	Tissue	Flank skin	0.75	0.91 (10/11)	Blood	0.86	0.86 (6/7)
		Eyelid skin	0.64		Spleen	0.5	
Red	Swab or blood	Arm swab	1.0	1.0 (7/7)	Rectal swab	0.5	0.67 (4/6)
		Eyelid swab	1.0		Blood	0.67	
	Tissue	Arm skin	1.0	1.0 (7/7)	Large intestine	1.0	1.0 (4/4)
		Eyelid skin	1.0		Small intestine	0.83	

It should be noted that the swab/blood samples appear to be superior to tissue samples in all instances apart from adenovirus in red squirrels when using the qPCR developed in this study

Both assays have led to novel findings that re-enforce the importance of sensitive molecular diagnostic techniques in wildlife disease surveillance studies, where the use of gross lesions and/or less sensitive molecular methods are likely to underestimate pathogen prevalence. While all the red squirrel cases suspected of having SQPV based on gross post-mortem findings were positive for SQPV when assayed using qPCR, an additional case of SQPV was discovered that had gastrointestinal pathology recorded as the cause of death. Similarly, gastrointestinal lesions have been reported in a single SQPV-infected red squirrel in Northern Ireland (Collins et al. 2014). Other SQPV-infected red squirrels in this study yielded SQPV in the gastrointestinal tract, with an increase in RQ passing through the gastrointestinal tract potentially indicating replication within the intestine. The oropharynx is also reported to be a site of replication in red squirrels (Fiegna et al. 2016).

Novel cases of SADV are reported in this study also. All three red squirrels that had died from causes other than skin lesions, gastrointestinal lesions or road causalities showed evidence of SADV infection. The two squirrels with severe pediculosis had SADV consistently identified in multiple tissue types, albeit at relatively low levels. The cause of death of these two squirrels was thought to be due to anaemia, secondary to parasitism (Duff et al. 2010; Larose et al. 2010; Simpson et al. 2013b). However, haemorrhage has been reported in 70 % (7/10) of case studies of SADV infection (Martinez-Jimenez et al. 2011). The prevalence of SADV in lice found on infected individuals was 80 % (4/5: 95 % CI, 30–99 %), which combined with the relatively high occurrence of viral DNA in blood (67 % (6/9); 95 % CI 31–91 %) may suggest a potential role of the parasite in pathogen transmission. Additionally, the red squirrel that had pneumonia also showed SADV DNA presence in all tissues analysed. The lung RQ value was the highest recorded in any infected individual (12.52), and, indeed, viral prevalence in lung tissue was relatively common in infected individuals (64 %, 7/11; 95 % CI, 32–88 %) (Fig. 3).

In conclusion, the use of appropriate chemistries and software allowed straightforward development of two multiplex assays that allow fast processing of samples while simultaneously producing more reliable results (and at a reduced cost) than published assays. Increased sensitivity had provided new information about the pathogenesis and epidemiology of the target pathogens. While these new assays have specific benefits for epidemiological investigations into the diseases of squirrels, it is hoped this work will have a wider impact by stimulating development of further multiplex qPCRs that will maximise information collected from investigations into multi-pathogen systems, which are becoming increasingly important as knowledge of co-infection dynamics increases (Telfer et al. 2010).

## Electronic supplementary material

Below is the link to the electronic supplementary material.

ESM 1 (DOCX 409 kb)
