# Supplementary material for: Enhancement of wildlife disease surveillance using multiplex quantitative PCR: development of qPCR assays for major pathogens in UK squirrel populations
Source: Eur J Wildl Res. 2016 Jul 28;62(5):589–99. doi: 10.1007/s10344-016-1031-z (PMC7088385; doi:10.1007/s10344-016-1031-z)
Supplement: Supplementary file 1 — (DOCX 409 kb) [file 10344_2016_1031_MOESM1_ESM.docx]

**Enhancement of wildlife disease surveillance using multiplex quantitative PCR: development of qPCR assays for major pathogens in UK squirrel populations**

Timothy D. Dale^1^, Julian Chantrey^1^, David Jones^2^, Phillip C. Watts^3^, Kieran Pounder^1^, David J. Everest^4^ and Michael E. Begon^1^

^1^Institute of Integrative Biology, University of Liverpool, Biosciences Building, Crown Street, Liverpool, L69 7ZB, UK.

^2^Institute of Infection and Global Health, University of Liverpool, UK.

^3^Department of Ecology, University of Oulu, FI-90014, Finland.

^4^Animal and Plant Health Agency, Weybridge, New Haw, Addlestone, Surrey KT15 3NB, UK.

Corresponding author: chantrey@liverpool.ac.uk

***S1:* Endogenous control sequence determination**

Primers where designed based on the thirteen-lined squirrel sequences identified during the BLASTn search using Primer3Plus software (Untergasser, Nijveen et al. 2007). Two primer pairs for each reference gene were selected for trials.

***Reaction conditions and cycle program***

The primers (Eurofins MWG Operon, Edersberg, Germany) were then tested using 10µl of Precision™ mastermix SYBR® green (2x concentration) (Primer Design, Southampton, UK), 200nM final concentration of each primer, 5µl of DNA template and the total volume made up to 20µl with molecular grade water. DNA templates were assessed for DNA concentration using a NanoDrop 1000 Spectrophotometer (Thermo Scientific, Asheville, United States) and diluted to 20ng/µl resulting in 100ng of DNA template in each reaction. A Lightcycler® 480 II real-time cycler (Roche, Welwyn Garden City, UK) was programmed with the following reaction conditions; enzyme activation occurred with a single cycle of 10 minutes at 95^o^C followed by 40 cycles consisting of denaturation for 15 seconds at 95^o^C, followed by annealing for 60 seconds at 52^o^C. A relatively low T_a_ was selected to encourage primer binding and facilitate the selection of primers with high specificity even at lower temperatures. The primers were tested against DNA templates extracted from mouse fibroblasts (originating from cell culture), cultured grey squirrel kidney cells, grey squirrel skin and blood, red squirrel skin and blood and avian hepatic tissue. All were extracted using a DNeasy® blood and tissue kit (Qiagen, Manchester, UK) following the manufacturer’s protocol. The resultant amplified products were then separated by agarose gel electrophoresis (3% agarose, 8 V/cm, 90 minutes). Those primers that showed a single band of approximately equal size to the predicted amplicon size by gel electrophoresis were then selected for sequencing.

***PCR product sequencing***

Excess primers and nucleotides were removed from the PCR products using the ExoSAP-IT® kit containing shrimp alkaline phosphotase and exonuclease (Affymetrix, High Wycombe, UK) following the manufacturer’s protocol. The BigDye® Terminator v3.1 Cycle Sequencing kit (Applied Biosystems, Life Technologies, Paisley, UK) was then used for the sequencing reaction using the PCR primers. One µl of the cleaned product was added to 9µl of reaction mix (5x sequencing buffer, 0.75µl BigDye® 3.1 (Applied Biosystems, Life Technologies, Paisley, UK) and 1.6pM of left or right primers). These then underwent 25 cycles; denaturation 96^o^C for 10 seconds, annealing 50^o^C for 5 seconds and extension 60^o^C for 4 minutes. This was then precipitated using 3M sodium acetate followed by re-suspension in HiDi™ formamide (Applied Biosystems, Life Technologies, Paisley, UK). The sequence of products was then determined using an ABI 3130xl (Applied Biosystems, Life Technologies, Paisley, UK).

Alignments of the determined sequence with the target sequence were performed in Mega 5.1 (Tamura, Peterson et al. 2011). Two candidate endogenous target genes were selected; those primer pairs that produced complementary sequences with each other and to the target region in both species of squirrel in both sample types (skin and blood).

***S2:* Calculation of DNA copies per standard oligonucleotide**

**Note**

mass of 1 molecule (g) = MW / Avogrado’s constant (6.02x10^23^).

To convert mass to moles

moles = mass/MW

**MPX A**

**Grey SQPV**

MW (g/mol) = 34496

Mass of 1 molecule = 34496/6.02x10^23^ = 5.73x10^-20^ g = 5.73x10^-8^pg

Therefore in 200pg there is 200/5.73x10^-8^= 3.49x10^9^copies

Number of pmol in 200pg = 200/34496 = 0.0058 pmol per reaction

Therefore want a stock solution of 0.0058pmol/5ul = 0.0012 pmol/ul

From 10pmol/ul solution need to dilute 1:8624.

Stock solution = 40pg/ul (200pg/5ul) = 6.981 x 10^8^ copies/ul

**Grey Adeno**

MW (g/mol) = 30204

Mass of 1 molecule = 30204/6.02x10^23^ = 5.02x10^-20^ g = 5.02x10^-8^pg

Therefore in 200pg there is 200/5.02x10^-8^= 3.99x10^9^copies

Number of pmol in 200pg = 200/30204 = 0.0066 pmol per reaction

Therefore want a stock solution of 0.0066pmol/5ul = 0.0013 pmol/ul

From 10pmol/ul solution need to dilute 1:7551.

Stock solution = 40pg/ul = 7.973 x 10^8^ copies/ul

**Grey PGK**

MW (g/mol) = 39627.7

Mass of 1 molecule = 39627.7/6.02x10^23^ = 6.58x10^-20^ g = 6.58x10^-8^pg

Therefore in 200pg there is 200/6.58x10^-8^= 3.04x10^9^copies

Number of pmol in 200pg = 200/39627.7= 0.0051 pmol per reaction

Therefore want a stock solution of 0.0051pmol/5ul = 0.0010 pmol/ul

From 10pmol/ul solution need to dilute 1:9907.

Stock solution = 40pg/ul = 6.077 x 10^8^ copies/ul

**MPX B**

**Red SQPV**

MW (g/mol) = 22020

Mass of 1 molecule = 22020/6.02x10^23^ = 3.66x10^-20^ g = 3.66x10^-8^pg

Therefore in 200pg there is 200/3.66x10^-8^= 5.47x10^9^copies

Number of pmol in 200pg = 200/22020 = 0.0091 pmol per reaction

Therefore want a stock solution of 0.0091pmol/5ul = 0.0018 pmol/ul

From 10pmol/ul solution need to dilute 1:5505.

Stock solution = 40pg/ul = 1.094 x 10^9^ copies

**Red Adeno**

MW (g/mol) = 34504

Mass of 1 molecule = 34504/6.02x10^23^ = 5.73x10^-20^ g = 5.73x10^-8^pg

Therefore in 200pg there is 200/5.73x10^-8^= 3.49x10^9^copies

Number of pmol in 200pg = 200/34504 = 0.0058 pmol per reaction

Therefore want a stock solution of 0.0058pmol/5ul = 0.0012 pmol/ul

From 10pmol/ul solution need to dilute 1:8626.

Stock solution = 40pg/ul = 6.979 x 10^8^ copies/ul

**Red PGK**

MW (g/mol) = 27296

Mass of 1 molecule = 27296/6.02x10^23^ = 4.53x10^-20^ g = 4.53x10^-8^pg

Therefore in 200pg there is 200/4.53x10^-8^= 4.41x10^9^copies

Number of pmol in 200pg = 200/27296 = 0.0073 pmol per reaction

Therefore want a stock solution of 0.0073 pmol/5ul = 0.0015 pmol/ul

From 10pmol/ul solution need to dilute 1:6824.

Stock solution = 40pg/ul = 8.822 x 10^8^ copies/ul

***S3:* Oligonucleotide serial dilutions for qPCR standard curves**

**Reagents**

1. Lambda DNA (500µg, 0.3µg/µl) (Thermo-Fisher, Loughborough, UK)
2. Oligonucleotide standards (<120bp - Eurofins MWG Operon, Edersberg, Germany, >120bp – Integrated DNA Technologies, Leuven, Belgium)
3. Molecular water (Thermo-Fisher, Loughborough, UK)

**Preparation**

1. Prepare 50x and 1x carrier (lambda DNA) solutions
   1. Dilute 7µg (23.33µl (0.3µg/µl) carrier DNA with 4.66µl water to give 50x carrier solution (250ng/µl) – vortex thoroughly.
   2. Dilute 6µl 50x solution with 244µl water to obtain 1x carrier solution (5ng/µl) – vortex thoroughly.
2. Dilute standards as below;

| **Standard** | **Dilution factor** | **Volume of standard (µl)** | **Volume of Water (µl)** | **Concentration (copies/µl)** |
| --- | --- | --- | --- | --- |
| SQPVA | 139.62 | 5 | 693 | 5 x 10^6^ |
| ADVA | 159.36 | 5 | 792 | 5 x 10^6^ |
| PGKA | 121.58 | 5 | 603 | 5 x 10^6^ |
| SQPVB | 218.6 | 5 | 1088 | 5 x 10^6^ |
| ADVB | 139.62 | 5 | 693 | 5 x 10^6^ |
| PGKB | 176.6 | 5 | 878 | 5 x 10^6^ |

1. Dilute solutions of 2 as below to create point 1 (1.0 x 10^6^ copies/µl)

| **Standard** | **Dilution factor** | **Volume std 1 (µl)** | **Volume std 2 (µl)** | **Volume std 3 (µl)** | **Volume carrier DNA (x50)** | **Volume water (µl)** |
| --- | --- | --- | --- | --- | --- | --- |
| uniplex | 5 | 20 | - | - | 2 | 78 |
| duplex | 5 | 20 | 20 | - | 2 | 58 |
| triplex | 5 | 20 | 20 | 20 | 2 | 38 |

1. Dilute 5µl point 1 solution with 45µl of carrier DNA (x1) to create point 2.
2. Repeat previous step four times to create dilution points 3-6.
3. Add 50ul of 1xcarrier to a tube marked negative control. Store all tubes at -20^o^C.

| **Dilution Point** | **Number of copies/µl** | **Number of copies/rx (10µl/rx)** |
| --- | --- | --- |
| 1 | 1000000 | 10000000 |
| 2 | 100000 | 1000000 |
| 3 | 10000 | 100000 |
| 4 | 1000 | 10000 |
| 5 | 100 | 1000 |
| 6 | 10 | 100 |
| 7 | 1 | 10 |
| Negative | 0 | 0 |

***S4:* qPCR reaction and plate template**

Date: Lab: Assay:

|  | **Type** | **Concentration** | **Concentration in reaction** | **Volume (µl)**  **per reaction** | **Total volume made up** |
| --- | --- | --- | --- | --- | --- |
| Master mix |  |  |  |  |  |
| SQPV L Primer |  |  |  |  |  |
| SQPV R Primer |  |  |  |  |  |
| SQPV Probe |  |  |  |  |  |
| Adeno L Primer |  |  |  |  |  |
| Adeno R Primer |  |  |  |  |  |
| Adeno Probe |  |  |  |  |  |
| PGK L Primer |  |  |  |  |  |
| PGK R Primer |  |  |  |  |  |
| PGK Probe |  |  |  |  |  |
| Water |  |  |  |  |  |
| Template |  |  |  |  |  |
|  |  |  | Total: |  |  |

|  |  |  |  |  |  |  |  |  |  |  |  |  |  |
| --- | --- | --- | --- | --- | --- | --- | --- | --- | --- | --- | --- | --- | --- |
|  |  | 1 | 2 | 3 | 4 | 5 | 6 | 7 | 8 | 9 | 10 | 11 | 12 |
|  | A | Sample 1 | Sample 1 | Sample 1 | NTC | Sample 2 | Sample 2 | Sample 2 | NTC | Sample 3 | Sample 3 | Sample 3 | NTC |
|  | B | Sample 4 | Sample 4 | Sample 4 | NTC | Sample 5 | Sample 5 | Sample 5 | NTC | Sample 6 | Sample 6 | Sample 6 | NTC |
|  | C | Sample 7 | Sample 7 | Sample 7 | NTC | Sample 8 | Sample 8 | Sample 8 | NTC | Sample 9 | Sample 9 | Sample 9 | NTC |
|  | D | Sample 10 | Sample 10 | Sample 10 | NTC | Sample 11 | Sample 11 | Sample 11 | NTC | Sample 12 | Sample 12 | Sample 12 | NTC |
|  | E | Sample 13 | Sample 13 | Sample 13 | NTC | Sample 14 | Sample 14 | Sample 14 | NTC | Sample 15 | Sample 15 | Sample 15 | NTC |
|  | F | Sample 16 | Sample 16 | Sample 16 | NTC | Sample 17 | Sample 17 | Sample 17 | NTC | Sample 18 | Sample 18 | Sample 18 | NTC |
|  | G | Sample 19 | Sample 19 | Sample 19 | NTC | Sample 20 | Sample 20 | Sample 20 | NTC | Sample 21 | Sample 21 | Sample 21 | NTC |
|  | H | Sample 22 | Sample 22 | Sample 22 | NTC | SQPV  +ve | SADV +ve | PGK +ve | NTC | Stan-  dard | Stan-  dard | Stan-  dard | Calib-  rator |

Time;

Temp;

Dissociation Y/N

Reps;

***S5:* Protocol (a)**

**Isolation of total DNA from Swabs (Qiagen DNeasy^®^ kit)**

This protocol is for isolation of total (genomic and mitochondrial) DNA from cotton or Dacron swabs latter preferred).

**Important points before starting**

- Perform all centrifugation steps at room temperature (15–25°C).

**Things to do before starting**

- Equilibrate Buffer AE or distilled water for elution to room temperature (15–25°C).
- Set a heated block to 56°C for use in step 3.
- If Buffer AL or Buffer ATL contains precipitates, dissolve by placing on heatblock at 56°C with gentle agitation.
- Ensure that Buffers AW1 and AW2 have been prepared according to the instructions on page 12 of the handbook.

**Procedure**

1. **Shorten shaft of swab if swab is unable to move in microtube. Using designated disposable forceps lift and break the shaft for dacron swabs (white shaft) or cut using scissors for cotton swabs (blue shaft).**

Make sure to wipe scissors with trigene inbetween samples.

**2. Add 20 μl proteinase K and 400 μl Buffer ATL, close the lid, and mix by pulse-vortexing for 10 s.**

**3. Place the tube in a heated block and incubate at 56°C for at least 1 h.**

Vortex the tube approximately every 10 minutes to improve lysis.

**Swabs**

**4. Briefly centrifuge the tube to remove drops from the inside of the lid.**

**5. Add 400 μl Buffer AL close the lid, and mix by pulse-vortexing for 15 s.**

To ensure efficient lysis, it is essential that the sample and Buffer AL are thoroughly mixed to yield a homogeneous solution. A white precipitate may form when Buffer AL is added to Buffer ATL. The precipitate does not interfere with the procedure and will dissolve during incubation in step 6.

**6. Briefly centrifuge the 2 ml tube to remove drops from the inside of the lid.**

**7. Add 200 μl ethanol (96–100%), close the lid, and mix by pulse-vortexing for 15 s.**

To ensure efficient binding in step 10, it is essential that the sample and ethanol are thoroughly mixed to yield a homogeneous solution.

**8. Briefly centrifuge the 2 ml tube to remove drops from the inside of the lid.**

**9. Using the forceps lift the swab and squeeze against the side of the tube. Briefly centrifuge tube to remove drops from the side of the tube.**

**10. Place swab upside down in a new 1.5ml microtube and centrifuge at 14,000rpm for 2min.**

**11. Carefully transfer the entire lysate from steps 9 and 10 to the DNeasy column (in a 2 ml collection tube) without wetting the rim (maximum 700µl), close the lid, and centrifuge at 6000 x g (8000 rpm) for 1 min. Empty collection tube and repeat for remaining lysate. Place the column in a clean 2 ml collection tube, and discard the collection tube containing the flow-through.**

If the lysate has not completely passed through the membrane after centrifugation, centrifuge again at a higher speed until the column is empty.

**12. Carefully open the column and add 500 μl Buffer AW1 without wetting the rim. Close the lid and centrifuge at 6000 x g (8000 rpm) for 1 min. Place the column in a clean 2 ml collection tube, and discard the collection tube containing the flow-through.**

**13. Carefully open the column and add 500 μl Buffer AW2 without wetting the rim. Close the lid and centrifuge at 20,000 x g (14,000 rpm) for 3 min to dry the DNeasy membrane. Place the column in a clean 1.5ml collection tube (not supplied), and discard the collection tube containing the flow-through.**

Contact between the column and the flow-through should be avoided. Some centrifuge rotors may vibrate upon deceleration, resulting in the flow-through, which contains ethanol, coming into contact with the column. Take care when removing the column and collection tube from the rotor, so that flow-through does not come into contact with the column.

**12. Carefully open the lid of the column. Apply 100 μl buffer AE directly onto the DNeasy membrane. Close the lid and incubate at room temperature for 1min.**

**13. Then centrifuge for 1 min at 6000x g (8000rpm).**

**14. Discard column and store elute as recommended in the handbook.**

***S5:* Protocol (b)**

**Isolation of total DNA from blood cell pellet (Qiagen FlexiGene DNA kit)**

**Things to do before starting;**

- IF using a new kit resuspend the lyophilized QIAGEN Protease in the following volumes of Buffer FG3 (hydration buffer):
  - 0.3 ml when using 50 ml FlexiGene DNA Kit
  - 1.4 ml when using the 250 ml FlexiGene DNA Kit.

Dissolved QIAGEN Protease should be stored at 2–8°C or in aliquots at

–20°C.

- Calculate the total volume of blood to be processed. For every 1 ml of

blood, mix together 600 μl Buffer FG2 (denaturation buffer) and 5 μl

reconstituted QIAGEN Protease (see Table 5). The Buffer FG2/QIAGEN Protease mixture should be prepared not more than 1 hour before use.

- Heat a heating block or water bath to 65°C for use in steps 5 and 13.
- Place Isopropanol and 70% Ethanol in the fridge.
- Table 5. Volumes of Buffer FG2 and QIAGEN Protease required for

different batch volumes;

| Total volume of blood in batch (ml) | 1 | 3 | 6 | 12 | 18 | 36 |
| --- | --- | --- | --- | --- | --- | --- |
| Volume of buffer FG2 (ml) | 0.6 | 1.8 | 3.6 | 7.2 | 10.8 | 21.6 |
| Volume of Qiagen Protease (µl) | 5 | 15 | 30 | 60 | 90 | 180 |

**Protocol for extraction of DNA from blood cell pellet (originating from 1ml whole blood).**

1. On ice place four 2mm stainless steel beads to the cell pellet in the microtube and agitate the bottom of the cell pellet with a pipette tip.
2. Add 1 ml Buffer FG1 to microtube contained cell pellet.
3. Place in Tissue Lyser machine. Shake for 30 oscillations/second for one minute.
4. Transfer sample to 5 ml preparation tube and add a further 1.5ml of FG1 buffer. Cap and vortex for 2-3 seconds.
5. Centrifuge for 5 min at 2000 x g in centrifuge.
6. Discard the supernatant and leave inverted on a clean sheet of absorbent paper for 2 mins, taking care that the pellet remains in the tube.

Note: In rare cases the pellet may be loose, so pour slowly. Inverting the tube onto absorbent paper minimizes backflow of supernatant from the rim and sides of the tube onto the pellet.

1. Add 0.6 ml Buffer FG2/QIAGEN Protease (see “Things to do before starting”), close the tube, and vortex immediately until the pellet is completely homogenized. Inspect the tube to check that homogenization is complete.

Note: When processing multiple samples, vortex each tube immediately after addition of Buffer FG2/QIAGEN Protease. Do not wait until buffer has been added to all samples before vortexing.

1. Invert the tube 3 times, place it in a heating block or water bath, and incubate at 65°C for 10 min.

Note: The sample changes color from red to olive green, indicating protein digestion.

1. Add 0.5 ml isopropanol (100%) and mix thoroughly by inverting 20 times until the DNA precipitate becomes visible as threads or a clump.
2. Centrifuge for 3 min at 2000 x *g*.

Note: If the resulting pellets are loose, centrifugation can be prolonged or a higher *g*-force can be used.

1. Discard the supernatant and briefly invert the tube onto a clean piece of absorbent paper, taking care that the pellet remains in the tube.

Note: In rare cases the pellet may be loose, so pour slowly. If the white blood cell count of the sample was sufficiently high, the DNA should be visible as a small white pellet.

1. Add 0.5 ml 70% ethanol and vortex for 5 s.
2. Centrifuge for 3 min at 2000 x *g*.

Note: If the resulting pellets are loose, centrifugation can be prolonged or a higher *g*-force can be used.

1. Discard the supernatant and leave the tube inverted on a clean piece of absorbent paper for at least 5 min, taking care that the pellet remains in the tube.

Note: In rare cases the pellet may be loose, so pour slowly. Inverting the tube onto absorbent paper minimizes backflow of ethanol from the rim and sides of the tube onto the pellet.

1. Air-dry the DNA pellet until all the liquid has evaporated (at least 5 min).

Note: Avoid over-drying the DNA pellet, since over-dried DNA is very

difficult to dissolve.

1. Add the required amount (see below) of Buffer FG3, vortex for 5 s at low speed, and dissolve the DNA by incubating for 1-2 hrs at 65°C in a heating block or water bath.

Note: If there is an obvious pellet then add 200µl of buffer FG3, so the pellet is only just detectable then add 100µl of buffer FG3. If no pellet is visible at all then add 50µl of buffer FG3.

Note: If the DNA is not completely dissolved, incubate the solution overnight at room temperature. If a reduced volume of Buffer FG3 is used, the incubation time may need to be prolonged.

This protocol is a modification of the Qiagen Flexigene whole blood extraction protocol with the addition of the homogenisation step detailed in Lundblom et al 2011.

**Reference**

Lundblom et al 2011. High-speed shaking of frozen blood clots for extraction of human and malaria parasite DNA. Malaria Journal. 10:229.

***S6* – Endogenous control primer trial.**

| **Gene** | **Primer set** | **Primer/Probe** | **Sequence** | **T_m_** | **Length** |
| --- | --- | --- | --- | --- | --- |
| ACTB | 3 | L1 | TACTGTACCTCTTCTAGACC | 57.6 | 121 |
|  | 3 | R1 | ACTCAGTAGAGAGACAACC | 57.5 |  |
|  | 5 | L2 | CATAACAGTACCTGAGACC | 59.2 | 121 |
|  | 5 | R2 | TACTCCATCAGACAGTCC | 59.9 |  |
| VIM | 2 | L1 | AGTAGCTATGTCCTCCAG | 58.9 | 107 |
|  | 2 | R1 | GACGTGTTAGGTAGTTCC | 59.1 |  |
|  | 5 | L2 | GGAACTACCTAACACGTC | 59.1 | 141 |
|  | 5 | R2 | GTGAGAAGTCTACCAGTC | 59.3 |  |
| PGK | 7 | L1 | TGTCTATGTGAATGATGC | 59.2 | 117 |
|  | 7 | R1 | GCAAAATAGTTCAACTCC | 59.6 |  |
|  | 4 | L2 | AGTAGAGAAAGCTTGTGC | 59.4 | 184 |
|  | 4 | R2 | CATCATTCACATAGACATC | 59.1 |  |
| GAPDH | 2 | L1 | GACCTCAACTACATCGTC | 59.6 | 185 |
|  | 2 | R1 | GAAGACATCAGTGGACTC | 59.2 |  |
|  | 3 | L2 | ATGGTGAGAGTCAGAGTG | 59.8 | 126 |
|  | 3 | R2 | GACGATGTAGTTGAGGTC | 59.6 |  |
| ALDOA | 1 | L1 | CTCTGTGAGATGGTCTTC | 59.5 | 203 |
|  | 1 | R1 | GGTTAGAAGTCGTGTGTC | 59.8 |  |
|  | 2 | L2 | AGTAGGAGAAGATACTCTGTG | 59.5 | 179 |
|  | 2 | R2 | AGGTAGGAAGAATATGACC | 59.8 |  |
| RPL27a | 1m | L1 | ACGTAGTGGTGTCCTAATTG | 60.9 | 139 |
|  | 1m | R1 | AGTGACTGGTTACAGGTG | 60.1 |  |
|  | 1 | L2 | GTACGTAGTGGTGTCCTAAT | 60.9 | 141 |
|  | 1 | R2 | AGTGACTGGTTACAGGTG | 60.1 |  |
|  | 6 | L3 | TAGGTCTGACTTCTTCTGG | 61.8 | 147 |
|  | 6 | R3 | GGACCCACTATAAACAGC | 62 |  |
| RPL9 | 1 | L1 | GGTACAAGATGAGGTCTG | 59.7 | 153 |
|  | 1 | R1 | GAGCTTGAGACACTGAAC | 59.9 |  |
|  | 2 | L2 | GTACAAGATGAGGTCTGTG | 59.8 | 152 |
|  | 2 | R2 | GAGCTTGAGACACTGAAC | 59.9 |  |
| RPS13 | 4 | L1 | CAACTTCAACTGTAGACTGC | 62.6 | 109 |
|  | 4 | R1 | ACGTTGTGGTACACTTAGG | 62.6 |  |
|  | 5 | L2 | CTAGCCACACTATGACTCC | 62.2 | 166 |
|  | 5 | R2 | GGAGAGTTCTACGAAAGC | 61.5 |  |
| RPL30 | 1 | L1 | CTCATGATACGGTACAACC | 62.9 | 162 |
|  | 1 | R1 | AAGTGGTCAGACAAGACC | 62.0 |  |
|  | 6 | L2 | GACCACAGGTAGTGATGTC | 62.4 | 102 |
|  | 6 | R2 | GTCTTAGTGGGCCTAGTTAC | 62.6 |  |
| LDHA | 4 | L1 | CAGTGGATTATCTTGACC | 59.8 | 109 |
|  | 4 | R1 | CTATCAGTTAACAGAACTGG | 59.1 |  |
|  | 4m | L2 | CAGTGGATTATCTTGACC | 59.8 | 112 |

***Table S6.1***. Primer sequences used to identify those candidate genes for use as a reference gene.

| **Reference Gene** | **Primer set** | **Squirrel species (G/R)** | **Single band (Y/N)** | **Predicted band size (bp)** | **Band size (bp)** |
| --- | --- | --- | --- | --- | --- |
| ALDOA | 1 | G | N | 203 | 400,550,600 |
|  |  | R | Y | 203 | 550 |
|  | 2 | G | N | 179 | 300,400,500 |
|  |  | R | Y | 179 | 200 |
| ACTB | 1 | G | Y | 121 | 70 |
|  |  | R | Y | 121 | 70 |
|  | 2 | G | Y | **121** | **120** |
|  |  | R | Y | **121** | **120** |
| GAPDH | 1 | G | Y | **185** | **180** |
|  |  | R | Y | **185** | **180** |
|  | 2 | G | Y | **126** | **150** |
|  |  | R | Y | **126** | **150** |
| LDHA | 1 | G | N | 109 |  |
|  |  | R | N | 109 |  |
|  | 2 | G | Y | **112** | **120** |
|  |  | R | Y | **112** | **120** |
| PGK | 1 | G | N | 117 |  |
|  |  | R | N | 117 |  |
|  | 2 | G | Y | **184** | **175** |
|  |  | R | Y | **184** | **175** |
| RPL9 | 1 | G | Y | **153** | **150** |
|  |  | R | Y | **153** | **150** |
|  | 2 | G | Y | **152** | **155** |
|  |  | R | Y | **152** | **155** |
| RPL27a | 1 | G | Y | **139** | **120** |
|  |  | R | Y | **139** | **120** |
|  | 2 | G | Y | 141 | 400 |
|  |  | R | N | 141 | 400, 450 |
| RPL30 | 1 | G | N | 162 | 300, 450 |
|  |  | R | Y | 162 | 300 |
|  | 2 | G | N | 102 | 100, 250 |
|  |  | R | N | 102 | 100, 300 |
| RPS13 | 1 | G | N | 109 | 200, 450 |
|  |  | R | Y | 109 | 550 |
|  | 2 | G | N | 166 | 200, 550 |
|  |  | R | N | 166 | 200, 550 |
| VIM | 1 | G | N | 107 | 120, 400, 550 |
|  |  | R | N | 107 | 120, 400, 1000 |
|  | 2 | G | N | 141 | 450, 600, 900 |
|  |  | R | Y | 141 | 250 |

***Table S6.2.*** Summary of PCR product size after initial trial of primers sets. Those sets that show a single band in the region of the predicted size are shown in bold. Those highlighted in grey are the candidate genes selected for sequencing.

Grey PGK target region –

AGTAGAGAAAGCTTGTGCCAATCCAGCAGCTGGTCTATTATCCTGTTGGAGAACCTTCACTTTCATATTGAGGAAGAAGGGAAGGAAATAGATGCTTCTGGGAACAAGGAAAGCCGAGTCAGCCGAAGTAGCTTTTCCGAGCTTCACTTTCCAAACCAGGGGATGTCTATGTGAATGATGA

Red PGK target region –

AGTAGAGAAAGCTTGTGCCAATCCTGCAGCTGGGTCTGTTATCCTGTTGGAGAACCTTCGCTTTCACGTGGAGGAAGAAGGGAAGGGAAAAGATRCTTCTGGGAACAAGGTGAAAGCCGAGCCAGCCAAAGTAGAAGCTTTCYGAGCTTCACTTTCCAAACTAGGGGATGTCTATGTGARTGATGA

Grey RPL9 target region –

TTGGTACAAGATGAGGTCTGTGTARGCTCACTTCCCCATCAACGTCTGTTATTCAGGAGAATGGGTCTCTTGTTGAAATCCGAAATTTCTTGGGTGAAAAATATATCCGCAGGGTTCRTATGAGGACAGGTGTTGCTTGTTCAGATGTCTCAAGCTC

Red RPL9 target region –

TTGGTACAAGATGTRGGTCTGTGTRMGCTCATYTTCCCCMTCACATGCKTGTTWTCTACAGGAGAATGGGTCTCTTGTTGAAATCCRAAATTTCTTGGGTGAAAATATATCCSCAGGGTTCGGWDKRGGACGGGYGTTGCTTGTTCAGTRTCTCAAGCTC

***Figure S6.1.*** Determined sequences of candidate reference genes for red and grey squirrels. SNPs are displayed in red using IUPAC nucleotide code. RPL 9 shows a large number of SNPs in red squirrels thus was discounted as a candidate for both species.

***S7:* Primer, probe and target sequences**

**Multiplex set A (grey squirrel)**

>SQPV_0520 G8R conserved hypothetical pox protein 55607:56389 forward

atgggtcttcgcataaaactcgaccggttgaggcagatagtgacgtacttttcggagttctcggaagaggtctccgtgaacgtcgacacggccgatgggctcatgtacatcttcgccgccctgggcggctccgtcaacatctgggcgatcgtgccgctcagcgccagcgtctgcttcgacggcgacaccaactgcgtgttcaacctcccggtgctcaaggtcaagaactgcctctgcagcttccacagcgacgccatgatctccatcgagccggacctcgaggcggacacggtgcgcctgaccagccaccacgtggtcagcgtggactgcaacaaggagtcgctgccccaccgcacggagacggacatctacctcaacatcgaccagaagaagtcgtacgtgttcaacttccacctctacgaggagaagtgctgtgggcgcaccatcatgaacctggagctgctcctgggcttcatcaagtgcatcagccagcaccagcacctgaccgtgagtttccgggacaagaacctggtgctctgcaccccggggaagcgggacaccttcacgcgggagtactccatgacggagtggagcgcggagctgcagaagtactccttccgcatggccatctcctcgctcaacaagctgcggggcttcaagaagcgcgtctccatgttcgagacgcgcgtggtcatggacgcggacgacaacatcatggggatgctcttcagcgaccgcatcgcctcgtaccgcgtcaacgtcttcatggccttcgaggattag

>gi|374713550|gb|JN205244.1| Adenovirus squirrel/GBR/SG-2012 isolate FS180/07 DNA polymerase gene, partial cds

ACCTTCATTGGAGTACTAACTGAACCAATCTATGTTTATGATATCTGTGGAATGTATGCCAGTGCTTTAACCCACCCCTTTCCTTCGGGCCAGCCTTTAGCACCCTACGACAGAAACATTGCTATCATACAGTGGCAAAACAAACTAAACAACCCCTCTCAGATAGATTATTTCAATACGGAATTACTTCCGGGAATCTTTACAATCGACGCTGATCCCCCTTCGGAAGAATGGACCGACACATTGCCACCTTTCTGCAGTCGAAAAGGAGGAAGACTAACATGGACAAATGAATCTCTGAGAGGGGAAATCTGCACGTCTATTGATATGATAACACTACATAATAGAAAATGGAAACTGAGAATAATTCCCGACGAAAGGTGTACGATTTTCCCCGAATGGAAATGCCTCTGCAAAGACTACGTGCAATTGAACATAAAGGCTAAAGAAAAAGCCGACGCGGCAAAAAACCAAACGGTGAGAAGTATAGCAAAACTTCTGTCC

>PGK grey

AGTAGAGAAAGCTTGTGCCAATCCAGCAGCTGGTCTATTATCCTGTTGGAGAACCTTCACTTTCATATTGAGGAAGAAGGGAAGGAAATAGATGCTTCTGGGAACAAGGAAAGCCGAGTCAGCCGAAGTAGCTTTTCCGAGCTTCACTTTCCAAACCAGGGGATGTCTATGTGAATGATGA

**Multiplex set B (red squirrel)**

>SQPV_0520 G8R conserved hypothetical pox protein 55607:56389 forward

atgggtcttcgcataaaactcgaccggttgaggcagatagtgacgtacttttcggagttctcggaagaggtctccgtgaacgtcgacacggccgatgggctcatgtacatcttcgccgccctgggcggctccgtcaacatctgggcgatcgtgccgctcagcgccagcgtctgcttcgacggcgacaccaactgcgtgttcaacctcccggtgctcaaggtcaagaactgcctctgcagcttccacagcgacgccatgatctccatcgagccggacctcgaggcggacacggtgcgcctgaccagccaccacgtggtcagcgtggactgcaacaaggagtcgctgccccaccgcacggagacggacatctacctcaacatcgaccagaagaagtcgtacgtgttcaacttccacctctacgaggagaagtgctgtgggcgcaccatcatgaacctggagctgctcctgggcttcatcaagtgcatcagccagcaccagcacctgaccgtgagtttccgggacaagaacctggtgctctgcaccccggggaagcgggacaccttcacgcgggagtactccatgacggagtggagcgcggagctgcagaagtactccttccgcatggccatctcctcgctcaacaagctgcggggcttcaagaagcgcgtctccatgttcgagacgcgcgtggtcatggacgcggacgacaacatcatggggatgctcttcagcgaccgcatcgcctcgtaccgcgtcaacgtcttcatggccttcgaggattag

>gi|374713550|gb|JN205244.1| Adenovirus squirrel/GBR/SG-2012 isolate FS180/07 DNA polymerase gene, partial cds

ACCTTCATTGGAGTACTAACTGAACCAATCTATGTTTATGATATCTGTGGAATGTATGCCAGTGCTTTAACCCACCCCTTTCCTTCGGGCCAGCCTTTAGCACCCTACGACAGAAACATTGCTATCATACAGTGGCAAAACAAACTAAACAACCCCTCTCAGATAGATTATTTCAATACGGAATTACTTCCGGGAATCTTTACAATCGACGCTGATCCCCCTTCGGAAGAATGGACCGACACATTGCCACCTTTCTGCAGTCGAAAAGGAGGAAGACTAACATGGACAAATGAATCTCTGAGAGGGGAAATCTGCACGTCTATTGATATGATAACACTACATAATAGAAAATGGAAACTGAGAATAATTCCCGACGAAAGGTGTACGATTTTCCCCGAATGGAAATGCCTCTGCAAAGACTACGTGCAATTGAACATAAAGGCTAAAGAAAAAGCCGACGCGGCAAAAAACCAAACGGTGAGAAGTATAGCAAAACTTCTGTCC

>PGK red

AGTAGAGAAAGCTTGTGCCAATCCTGCAGCTGGGTCTGTTATCCTGTTGGAGAACCTTCGCTTTCACGTGGAGGAAGAAGGGAAGGGAAAAGATRCTTCTGGGAACAAGGTGAAAGCCGAGCCAGCCAAAGTAGAAGCTTTCYGAGCTTCACTTTCCAAACTAGGGGATGTCTATGTGARTGATGA

***Figure S7.1.*** Multiplex sets A and B shown within target sequences. XX – left primer, XX – hydrolysis probe and XX – right primer (reverse sequence).


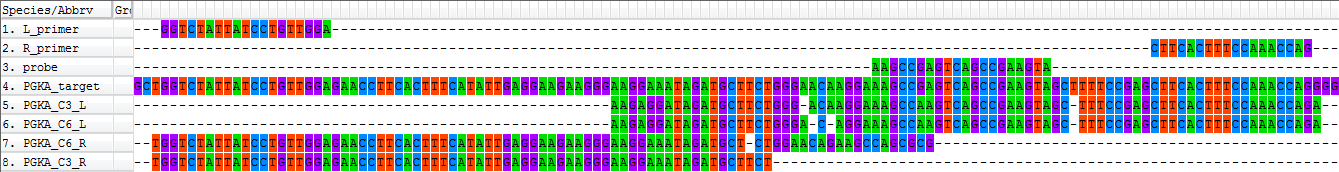


***Figure S7.2.*** Sequence alignment of PGK A PCR products.


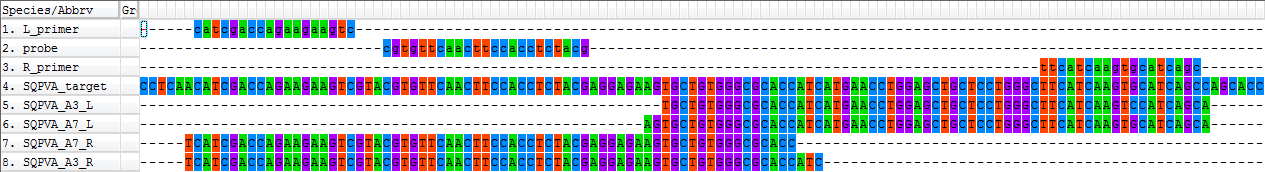


***Figure S7.3.*** Sequence alignment of SQPV A PCR products.


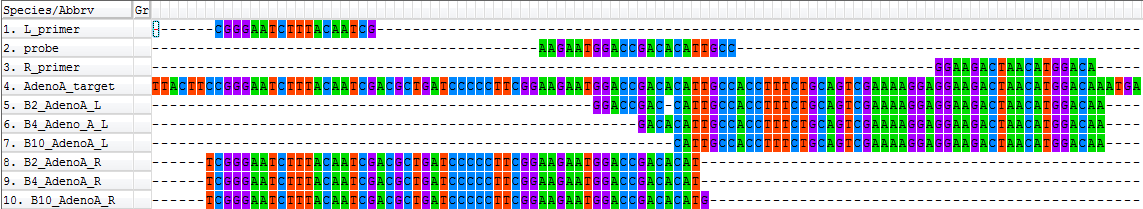


***Figure S7.4.*** Sequence alignment of SADV A PCR products.


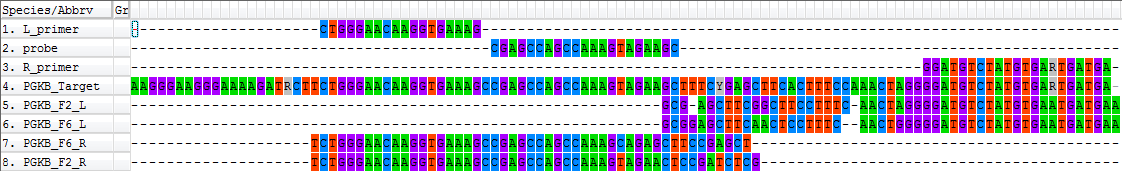


***Figure S7.5.*** Sequence alignment of PGK B PCR products.


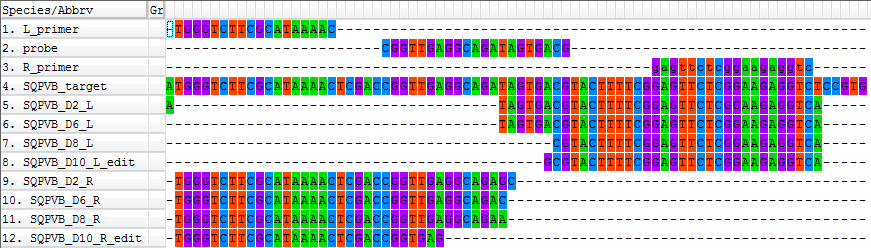


***Figure S7.6.*** Sequence alignment of SQPV B PCR products.


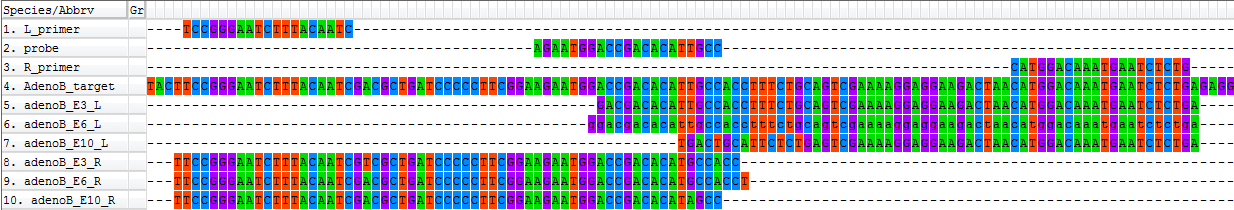


***Figure S7.7.*** Sequence alignment of SADV B PCR products.

***S8:* Assay optimisation**


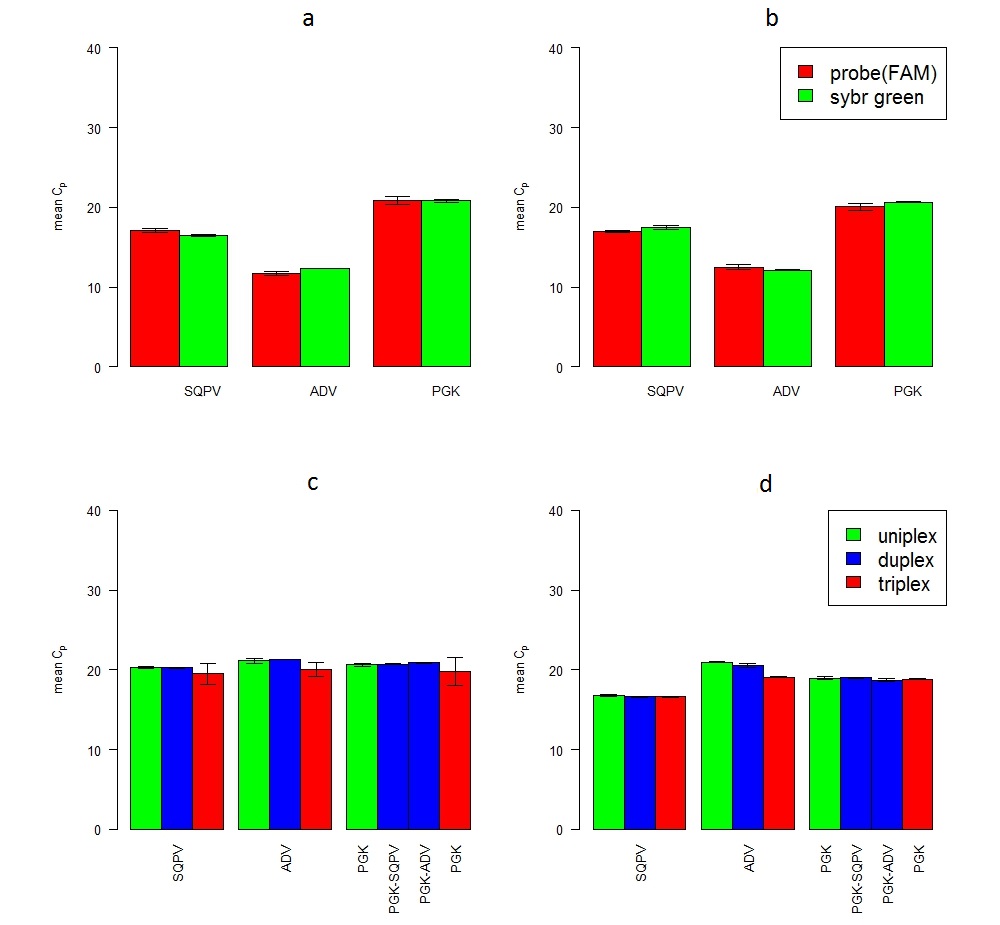


***Figure S8.1.*** Comparison of C_p_ values obtained; using SYBR® green and hydrolysis probe technology for multiplex sets for grey squirrels (a) and red squirrels (b) and using hydrolysis probes in uniplex, duplex and triplex for multiplex sets for grey squirrels (c) and red squirrels (d). All showed comparable values, with little indication of any negative influence of using hydrolysis probes and their use in multiplex reactions.


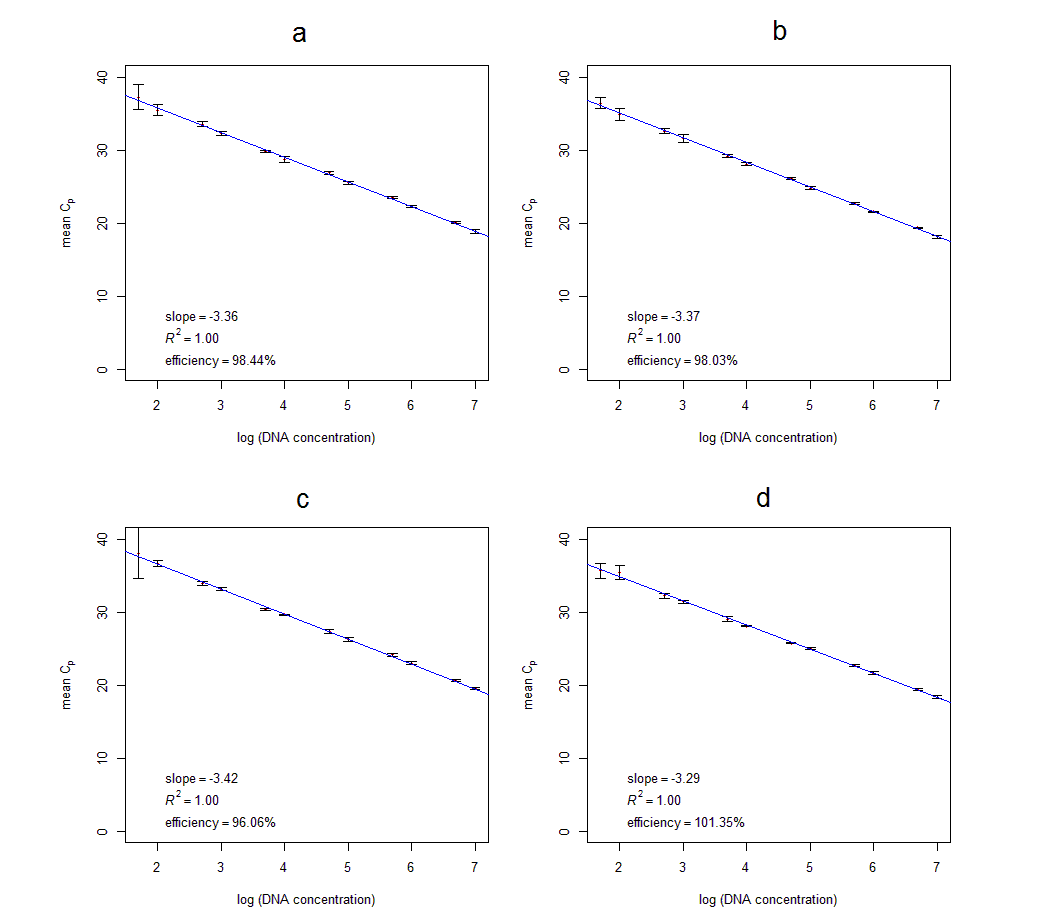


***Figure S8.2.*** Standard curves produced in duplex PCR for the grey multiplex set (using singleplex optimised primer and probe concentrations); PGK with SQPV as target (a), SQPV (b), PGK with ADV as target (c) and ADV (d). All assays display satisfactory efficiencies; 90-110%.


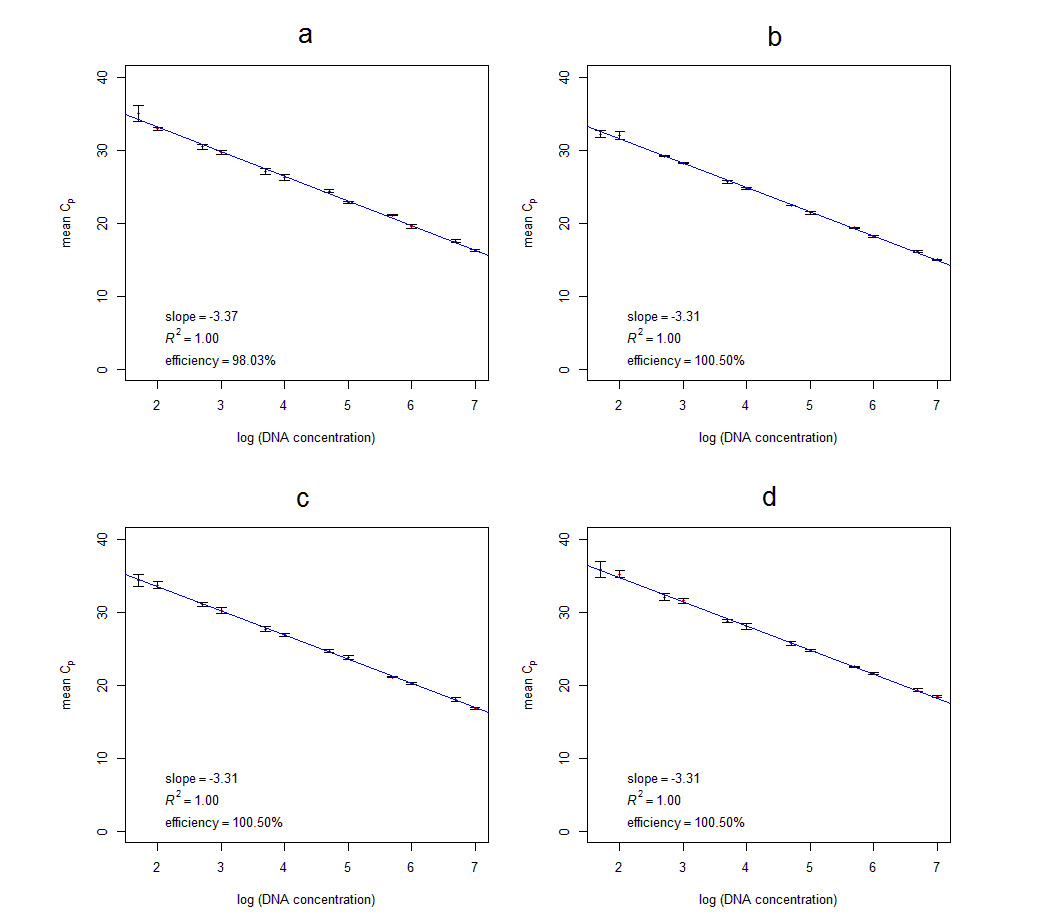


***Figure S8.3.*** Standard curves produced for duplex PCR for the red squirrel multiplex set (using optimised primer and probe concentrations); PGK with target SQPV gene (a), SQPV (b), PGK with target ADV gene (c) and ADV (d). All assays display satisfactory efficiencies; 90-110%.

***S9:* Assay validation**


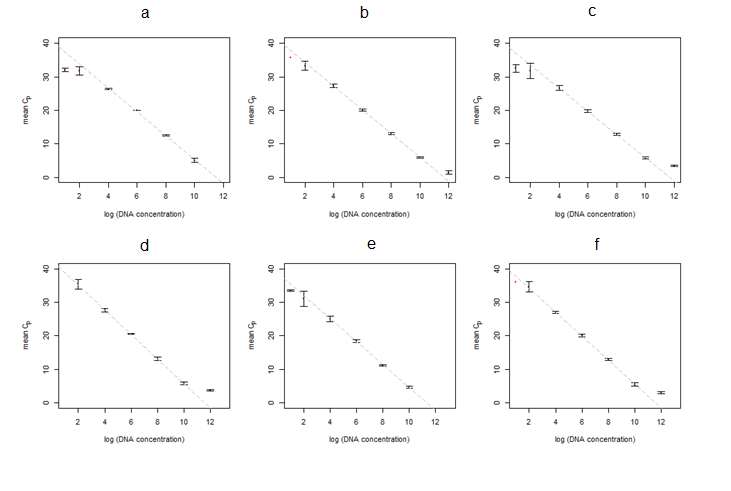


***Figure S9.1.*** Linear range of multiplex set for grey squirrels; PGK (a), SQPV (b) and SADV (c) and set for red squirrels; PGK (c), SQPV(d) and SADV (e). The dashed grey line indicates line of best fit based on data from DNA concentrations from 1.0 x10^3^ to 1.0x10^10^ copies per reaction.

***S10:* Post mortem assay trial**


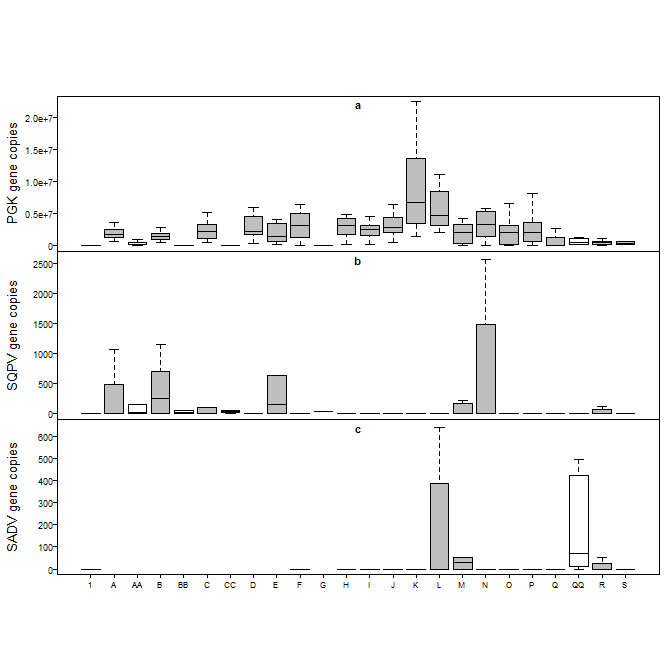


***Figure S10.1.*** Grey squirrel absolute quantification values obtained as a function of sample type for the reference gene PGK (a), SQPV (b) and SADV (c). Sample types along the x axis are defined as follows; 1 – lice, A – lip skin, AA – oral swab, B – eyelid skin, BB – eyelid swab, C – arm skin, CC – arm swab, D – chest skin, E - flank skin, F - submandibular lymph node, G – thymus, H – lung, I - heart, J – liver, K – kidney, L - spleen, M – blood, N – stomach, O – small intestine, P – large intestine, Q – faeces, QQ – rectal swab, R – skeletal muscle, S - brain.


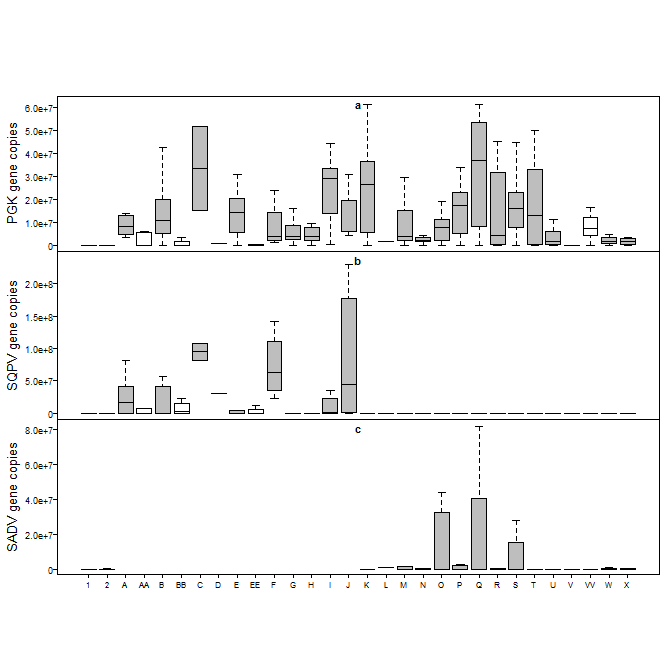


***Figure S10.2.*** Absolute quantification values obtained as a function of sample type for the reference gene PGK (a), SQPV (b) and SADV (c). Sample types along the x axis are defined as follows; 1 – lice, 2 – fleas, A – lip skin, AA – oral swab, B – eyelid skin, BB – eye swab, C – ear skin, D - nose skin, E - arm skin, EE – arm swab, F – digit skin, G – chest skin, H - flank skin, I – inguinal skin, J – gential skin, K - submandibular lymph node, L - thymus, M – lung, N - heart, O – liver, P – kidney, Q - spleen, R – blood, S – stomach, T – small intestine, U – large intestine, V – faeces, VV – rectal swab, W – skeletal muscle, X - brain.
